# The performance evaluation of NIPT for fetal chromosome microdeletion/microduplication detection: a retrospective analysis of 68,588 Chinese cases

**DOI:** 10.3389/fgene.2024.1390539

**Published:** 2024-06-07

**Authors:** Shichun Shen, Haimei Qi, Xianping Yuan, Jinhui Gan, Junkun Chen, Jungao Huang

**Affiliations:** ^1^ Department of Medical Genetics, Ganzhou Maternal and Child Health Hospital, Ganzhou, China; ^2^ Clinical Laboratory, Ganzhou Maternal and Child Health Hospital, Ganzhou, China; ^3^ Obstetrical Department, Ganzhou Maternal and Child Health Hospital, Ganzhou, China

**Keywords:** noninvasive prenatal testing, chromosome microdeletion/duplication, copy number variants, positive predictive value, cell-free fetal DNA

## Abstract

**Background:**

Chromosomal abnormalities are the main cause of birth defects in newborns. Since the inception of noninvasive prenatal testing (NIPT) technology, it has primarily been applied to the detection of common trisomy (T21, T18, T13). However, the application of NIPT in microdeletion and microduplication detection is still controversial.

**Methods:**

This study retrospectively analyzed the data of 68,588 cases that underwent NIPT at Ganzhou Maternal and Child Health Hospital in China. These data were used to evaluate the performance of NIPT in fetal chromosome microdeletion/microduplication detection and to investigate the key factors affecting the NIPT performance.

**Results:**

A total of 281 cases (0.41%) had positive NIPT results with copy number variants (CNVs), of which 161 were validated by karyotyping and chromosome microarray analysis (CMA). Among the 161 cases, 92 were confirmed as true positives through karyotyping or CMA, including 61 microdeletion cases and 31 microduplication cases, resulting in a positive predictive value (PPV) of 57.14%. Improvements in library construction methods increased the fraction of cell-free fetal DNA (cffDNA) from 13.76% to 18.44%, leading to a significant improvement in the detection rate (0.47% vs. 0.15%) and PPV (59.86% vs. 28.57%) of NIPT for CNVs.

**Conclusion:**

This study proved the robust performance of NIPT for fetal chromosome microdeletion/microduplication detection. In addition, the cffDNA fraction is a key factor influencing NIPT, with increased cffDNA fraction improving the performance of NIPT.

## 1 Introduction

Chromosomal structural abnormalities primarily encompass subchromosomal abnormalities, including duplications, deletions, inversions, translocations, etc. Chromosome microdeletion/microduplication, also known copy number variants (CNVs), can be classified into five clinical categories: pathogenic, likely pathogenic, variants of uncertain significance (VUS), likely benign, benign (Riggs et al., 2020). Pathogenic CNVs may give rise to a spectrum of phenotypes characterized by physical impairment, structural abnormalities, and intellectual disability. Some specific pathogenic CNVs can result in a typical clinical phenotype known as microdeletion/microduplication syndromes (MMs) (Wang et al., 2022). CNVs account for approximately 24% of congenital disorders and represent the second-largest contributor to congenital disorders after structural anomalies (Avram et al., 2021). The National Institute of Child Health and Human Development (NICHD) study reported a prevalence of 1.8% for pathogenic CNVs and 0.9% for VUS (Levy and Wapner, 2018).

Since the discovery of circulating fetal DNA in maternal plasma in 1997 (Lo et al., 1997), the detection of fetal genetic material from maternal blood samples has become an important method for screening fetal abnormalities. Noninvasive prenatal testing (NIPT), a massively parallel sequencing analysis of cell-free fetal DNA (cffDNA) in maternal plasma using next-generation sequencing (NGS) technology, has rapidly gained global popularity since it was applied commercially in 2011. Currently, the favorable detection performance of NIPT in screening for chromosome aneuploidy (CA) has been widely demonstrated (Petersen et al., 2017; Suo et al., 2023; Yang et al., 2023), but its performance in CNVs detection remains questionable (Chen et al., 2019).

In this study, we retrospectively analyzed 68,588 NIPT results at our center to evaluate the performance of NIPT in screening for CNVs and to analyze the main factors affecting the efficacy of NIPT in detecting CNVs. Our findings contributed clinical value to the application of NIPT in CNVs screening.

## 2 Materials and methods

### 2.1 Participant recruitment

This study recruited 68,592 pregnant women who underwent NIPT from April 2015 to June 2023 at Ganzhou Maternal and Child Health Hospital in China. Among them, 4 cases experienced multiple test failures due to low cffDNA fraction, resulting in a failure rate of 0.006%. Therefore, the total sample size included in this study was 68,588 cases. All subjects in this study were informed about the test methodology, the diseases covered in the screening, limitations and risks. They signed an informed consent form, and all methods were performed in accordance with relevant guidelines and regulations.

### 2.2 Sample preparation and sequencing

For each pregnant woman, 5 mL (EDTA anticoagulated) or 5–10 mL (free nucleic acid transport preservation tubes) of venous blood was collected. Maternal plasma was initially separated using an Eppendorf 5810R (Eppendorf, Germany) at 1,600 × *g* and 4°C, and the maternal supernatant plasma was obtained by further centrifugation at 16,000 × *g* and 4°C.

Free nucleic acids were extracted from plasma samples using the QIAamp DSP DNA Blood Mini Kit (Qiagen). The extracted DNA underwent end repair with the Ion Plus Fragment Library Kit (Life Technologies), and magnetic beads were employed to screen for DNA fragments smaller than 230 bp, enriching cffDNA. Subsequently, the enriched cffDNA was junction ligated and subjected to PCR amplification.

The prepared DNA libraries were quantified for concentration using RT-PCR, and the library concentrations were recorded. Dilutions were made based on the library concentration to ensure that all samples were sequenced at approximately the same concentration. The various diluted libraries were pooled into a total library and then subjected to NGS using the Bioelectron-seq 4000 sequencing platform (CFDA registration permit NO. 20153400309, CapitalBio, China). Sequencing of samples was performed to a maximum of 320 flows to generate raw data, with the mean length of the reads being approximately 135 base pairs (bp).

### 2.3 Sequencing data analysis

After sequencing, the sequencing data were analyzed using NIPT data analysis management software (CapitalBio Genomics, China) to obtain the Z-score of the sample chromosomes. The core algorithm for data analysis employed a general function to calculate the Z-score.

Raw data were filtered based on the following criteria: the mean length of the reads was >100 bp, the sequencing quality value (Q20) was >50%, the GC concentration was between 38% and 45%, and the fraction of cffDNA was ≥4%. The clean reads were aligned to the human reference genome (hg19) and filtered low-quality alignments and duplicates to obtain unique reads. As a result, a minimum of 3.5 million unique reads were obtained for each sample.

All chromosomes were first divided into segments with a bin size of 20 kb. Subsequently, the unique reads of each chromosome in each sample were calculated as a percentage of the unique reads of all autosomes in that sample, known as the reads ratio value (%chrN). The %chrN was calculated using the following equation:

$$\%\text{chrN}=\frac{\text{The}\;\text{total}\;\text{number}\;\text{of}\;\text{unique}\;\text{reads}\;\text{on}\;\text{chromosome}\;N}{\text{The}\;\text{total}\;\text{number}\;\text{of}\;\text{unique}\;\text{reads}\;\text{on}\;\text{all}\;\text{autosomes}}\times 100\%\;\left(N=1,2,3\ldots 22,X,Y\right)$$

Finally, the Z-score of the chromosome being tested was calculated using the following equation:

$$Z-\text{score}=\frac{\%\text{chrN}\;\text{of}\;\text{the}\;\text{sample}-\text{Mean}\;\text{of}\;\%\text{chrN}\;\text{of}\;\text{the}\;\text{reference}\;\text{sample}}{\text{Standard}\;\text{deviation}\;\text{of}\;\%\text{chrN}\;\text{of}\;\text{the}\;\text{reference}\;\text{sample}}$$

Each chromosome with an absolute value of the Z-score greater than three was marked with chromosome aneuploidies or microdeletions/microduplications; more details can be found in the references (Liao et al., 2014; Hu et al., 2016).

### 2.4 Prenatal diagnosis and pregnancy follow-up

Genetic counseling was provided to all positive cases. Those who opted for prenatal diagnosis underwent karyotyping using G-band resolution (400 bands) and chromosome microarray analysis (CMA) using CytoScanTM 750K (Affymetrix, United States) after obtaining samples via invasive amniocentesis. Individuals who declined prenatal diagnosis were followed up for subsequent pregnancy outcomes, and both prenatal diagnosis results and pregnancy outcomes were collected. Pregnant women with negative results were recommended routine prenatal tests and visits, with telephone follow-up 3–6 months after the expected date of delivery, in accordance with national guidelines. Neonatal follow-up focused on identifying any newborns with CNVs, and further genetic diagnosis was recommended if parents reported birth defects in the newborn.

### 2.5 Statistical analysis

Based on the NIPT results and prenatal diagnosis results, as well as the follow-up on pregnancy outcomes, the positive predictive value (PPV) was calculated using the formula PPV = TP/(TP + FP), where TP and FP represent the number of true positives and false positives, respectively. Since CNVs may exhibit normal postnatal follow-up due to insignificant symptoms, and we were unable to validate CMA in all tested newborns, the number of true negatives and false negatives could not be determined. Therefore, we were unable to calculate the negative predictive value, sensitivity, and specificity in this study. All data analyses were performed using SPSS 27.0 statistical software. Measured data were expressed as mean ± standard deviation (SD), and count data were expressed as percentage (%).

## 3 Results

### 3.1 Demographic characteristics of pregnant women

The maternal age ranged from 14 to 54 years old, with a mean age of 30.6 ± 5.77 years. Height ranged from 115 to 183 cm, with a mean height of 158.2 ± 5.03 cm. Weight varied from 30 to 103 kg, with a mean weight of 56.2 ± 8.35 kg. Body Mass Index (BMI) ranged from 12.98 to 35.86, with a mean BMI of 22.5 ± 3.12. The gestational age (GA) ranged from 12^+0^ to 36^+5^ weeks, with a mean GA of 17.2 ± 2.99 weeks. According to different clinical indications, the study population was categorized into six groups based on five common risk factors and other factors: advanced maternal age (AMA) with age ≥ 35 years, positive serum screening with high or critical risk of serum screening and MOM abnormality, Nuchal Translucency (NT) thickening with NT ≥ 2.5 mm, abnormal ultrasound soft indexes including echocardiograms, choroid plexus cysts, single umbilical artery, etc., adverse reproductive history with previous adverse pregnancy outcome, and other factors without the above five risk factors (Table 1).

**TABLE 1:** Demographics and clinical characteristics of pregnancies.

Characteristic	Number	Percent	mean ± SD
Maternal age (years)			
<20	1130	1.73%	30.6 ± 5.77
20–29	28507	43.55%	
30–34	17436	26.74%	
35–39	14289	21.75%	
≥40	4166	6.23%	
Height (cm)			
<150	1135	1.80%	158.2 ± 5.03
150–159	36048	56.86%	
160–169	25283	39.91%	
≥170	894	1.43%	
Weight (kg)			
<40	312	0.50%	56.2 ± 8.35
40–49	12978	20.70%	
50–60	29550	47.14%	
61–69	15387	24.55%	
≥70	4459	7.11%	
BMI			
<18.5	5143	8.30%	22.5 ± 3.12
18.5–20.9	16653	26.87%	
21–23.9	22446	36.21%	
24–27.9	14397	23.23%	
28–31.9	2901	4.68%	
≥32	446	0.72%	
GA (weeks)			
12^+0^–15^+6^	19029	29.35%	17.2 ± 2.99
16^+0^–19^+6^	37766	57.55%	
20^+0^–23^+6^	5944	8.95%	
24^+0^–27^+6^	2294	3.45%	
≥28^+0^	464	0.71%	
Type of pregnancy			
singleton pregnancy	62565	95.54%	
twin pregnancy	2956	4.46%	
Clinical indications			
AMA	19744	30.05%	
Positive serum screening	23369	35.56%	
NT thickening	742	1.18%	
abnormal ultrasound soft indexes	2360	3.59%	
adverse reproductive history	2065	3.07%	
other factors^a^	17250	26.54%	

^a^ If a pregnant woman had two or more of the five common risk factors, the first risk factor was determined based on the following order of priority: advanced age > positive serum screen > NT, thickening > abnormal soft ultrasound index > adverse maternal history.

### 3.2 Positive rates (PR) and characterization of positive cases

**FIGURE 1:**
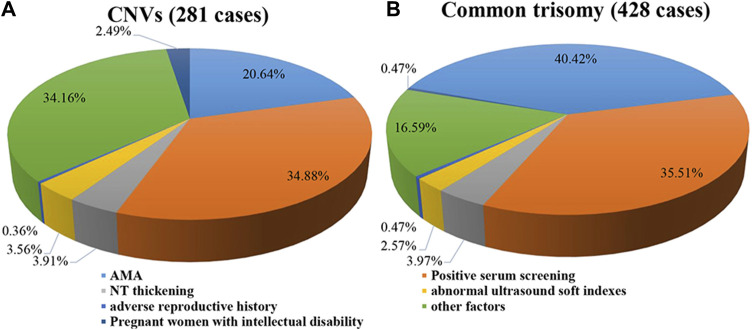
Clinical indications of cases with positive results. **(A)** Pie plot for CNVs. **(B)** Pie plot for common trisomy. Some of the cases with positive results were found to have intellectual disability themselves. Therefore, these pregnant women were classified in a separate category.

A total of 281 positive results with CNVs were detected in 68,588 samples (Supplementary Table S1), with a PR of 0.41% (281/68,588), including 193 cases with deletion CNVs and 88 cases with duplication CNVs. After comparing the clinical characteristics between the positive cases with CNVs and the positive cases with common trisomies, 65.84% of the positive CNVs cases exhibited clear clinical indications (AMA, positive serum screening, NT thickening, abnormal ultrasound soft indicators, and adverse reproductive history), and 34.16% showed no obvious clinical indications (Figure 1A). In contrast, among the positive cases for common trisomies, 83.41% had clear clinical indications, while 16.59% showed no obvious clinical indications (Figure 1B). The statistical analysis result indicated that the proportion of individuals with clear clinical indications among common trisomies positive cases was significantly higher than among CNV positive cases (Supplementary Table S2). In addition, 7 (2.49%) pregnant women with positive CNVs results were found to have intellectual disability themselves, five of whom were identified with CNVs during maternal verification, and 2 cases were confirmed to have the same CNVs as their mothers after prenatal diagnosis.

The proportion of advanced maternal age in positive CNVs cases was only 20.64%, while the proportion among positive common trisomy cases was as high as 40.42%. The mean age of positive CNVs cases was 29.08 ± 5.69 years, which was lower than the mean age of positive chromosome aneuploidy cases (31.87 ± 6.34 years), and also lower than the mean age of negative cases (30.59 ± 5.77 years), with statistically significant differences among them (Supplementary Figure S1).

### 3.3 Prenatal diagnosis of positive cases and PPV

Prenatal diagnosis was performed in 161 cases out of 281 positive results, giving a prenatal diagnosis rate of 57.30% (161/281). Of the 161 cases that underwent prenatal diagnosis 109 were deletions and 52 were duplicates, 61 deletions and 31 duplicates were diagnosed by CMA, giving a total PPV of 57.14% (92/161) (Table 2). PPV was 55.96% for microdeletion cases and 59.62% for microduplication, which were not statistically different (Supplementary Table S3). PPV was 54.76% in cases with CNVs size of 0–5 Mb, 60.00% in cases with sizes of 5–10 Mb, and 70.59% in cases with sizes of 10–20 Mb, showing a tendency of higher PPV with larger abnormal fragments, while PPV decreased to 50.00% when the abnormal fragment was ≥20 Mb (Supplementary Figure S2). In addition, we found that 22 out of 34 positive cases around 1 Mb were confirmed by CMA with a PPV of 64.71%.

**TABLE 2:** The detection efficiency of different CNVs size in NIPT.

Size (Mb)	Prenatal diagnostic validated by CMA								
	Deletion			Duplication			Total		
	Positive	Negative	PPV	Positive	Negative	PPV	Positive	Negative	PPV
0–5	31	30	50.82%	15	8	65.22%	46	38	54.76%
5–10	17	11	60.71%	7	5	58.33%	24	16	60.00%
10–20	8	1	88.89%	4	4	50.00%	12	5	70.59%
≥20	5	6	45.45%	5	4	55.56%	10	10	50.00%
Total	61	48	55.96%	31	21	59.62%	92	69	57.14%

### 3.4 Follow-up of pregnancy outcome

**FIGURE 2:**
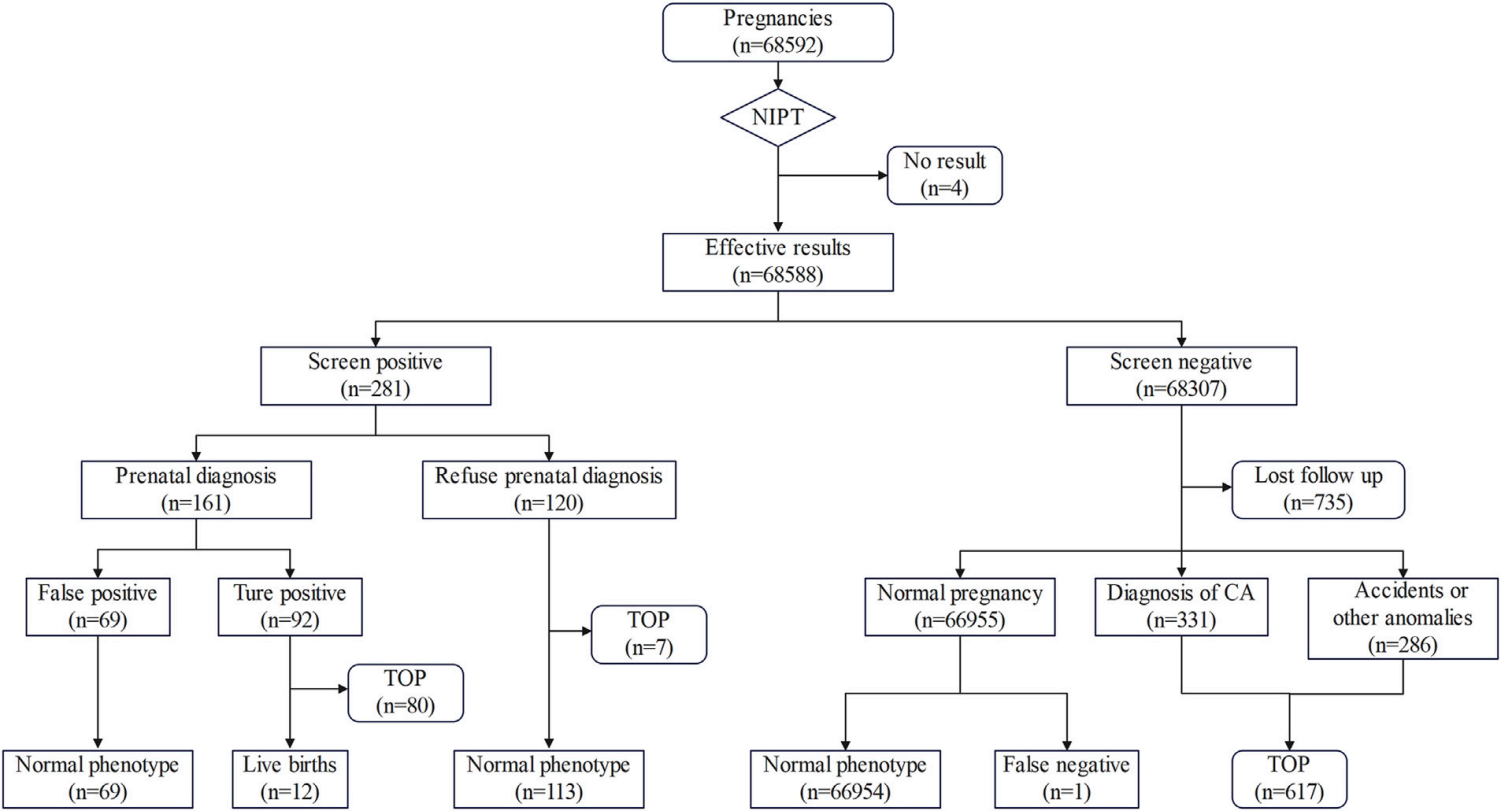
Flowchart of NIPT results and pregnancy outcomes. TOP, termination of pregnancy; CA, chromosome aneuploidy.

Follow-up results showed 80 of the 92 confirmed cases who underwent prenatal diagnosis chose to terminate their pregnancies, 12 chose to continue their pregnancies, and 69 negative cases chose to continue their pregnancies. In addition, seven of the 120 positive samples who did not undergo prenatal diagnosis chose to terminate their pregnancies because of ultrasound anomalies or for other reasons, and 113 had normal pregnancies and delivered normal newborns. The follow-up results of 68,307 negative cases showed 735 were lost due to refusal or inability to contact them, 286 were lost due to accidents or other anomalies, 331 were induced due to diagnosis of CA, and 66,954 showed no abnormality, with one false-negative finding. This false-negative case was diagnosed as Cri-du-Chat syndrome due to typical clinical signs. The outcomes of all pregnancies were shown in Figure 2.

### 3.5 CffDNA fraction affected NIPT test results

The cffDNA fraction is influenced by gestational age and enrichment technology. A low cffDNA fraction can lead to detection failure or produce false-negative results. In early 2018, we enhanced the assay by screening free DNA fragments below 230 bp during library construction, which contributed to an increased the cffDNA fraction in the assay data. The cffDNA fraction after improvement (18.44% ± 5.80%) showed statistical significance compared with the cffDNA fraction before improvement (13.76% ± 5.68%). In addition, there were no statistically significant differences in Reads (representing the sequencing throughput) and GC concentration, as shown in Supplementary Table S4. This result suggested that improving the cffDNA fraction can increase fetal sequencing data without increasing the sequencing Reads. Following the improvement, the PR and PPV of CNVs detected by NIPT increased significantly, as reflected in Table 3. These results suggested that the improved of cffDNA fraction significantly enhanced CNV detection.

**TABLE 3:** Comparison of Screening PR and PPV of prenatal diagnosis before and after the improvement.

Index		Crosstabulation				Chi-square tests		
		Positive	Negative	Total	Rate	Value	df	Asymptotic Sig
Screening PR	pre-improvement	19	12866	12885	0.15%	26.74	1	<0.001
	post-improvement	262	55441	55703	0.47%			
	Total	281	68307	68588	0.41%			
PPV of prenatal diagnosis	pre-improvement	4	10	14	28.57%	5.11	1	0.024
	post-improvement	88	59	147	59.86%			
	Total	92	69	161	57.14%			

Chi-Square tests were conducted using the Pearson Chi-Square, and the rate difference was found to be significant at the 0.05 level.

Therefore, we reviewed the previous false-negative result to explore whether the improvement of cffDNA fraction could rectify the false-negative result. We re-examined the retained plasma samples to reconstruct the library and re-sequenced the original library with triple Reads. The result of the triple-Reads review remained negative, but the reassessment of the re-based library revealed a positive result (Supplementary Table S5). We found that the sample in this case was obtained before the implementation of the improved library construction method in 2018. The cffDNA fraction during the initial test in 2017 was 11.70%, and after the reconstruction of the library using the new method, the cffDNA fraction increased to 18.02%, representing a 54.02% increase from the initial test. This result highlighted the importance of cffDNA fraction in NIPT. Therefore, the cffDNA fraction significantly impacted the performance of NIPT. Improvement of enrichment methods is necessary in clinical practice to enhance the cffDNA fraction.

## 4 Discussion

Since the inception of NIPT technology, it has been widely accepted for prenatal screening of common trisomy including T21, T18, and T13. Many studies have demonstrated the good detection performance of NIPT for T21, T18, and T13 screening (Petersen et al., 2017; Chen et al., 2019; Ma et al., 2023). In recent years, numerous studies have also reported that NIPT exhibited good detection performance for screening sex chromosome abnormalities (SCAs) (Xu et al., 2019; Lu et al., 2021; Luo et al., 2021).

The use of NIPT technology for CNVs screening has been highly controversial. In the absence of rigorous clinical validation, the American College of Obstetricians and Gynecologists (ACOG) and the Society for Maternal-Fetal Medicine (SMFM) currently do not recommend cffDNA microdeletions as a routine screening test for low-risk obstetrics populations due to the low PPV (Gregg et al., 2016). However, the ultimate goal of pregnancy for most Chinese parents is to have a healthy child, which is seen as the guarantee of a happy life (Li et al., 2017). These parents believe that a disability could result in a lower quality of life for the child, and they seek to be aware of any conditions affecting their child’s health during pregnancy (Ngan et al., 2020). Therefore, the application of NIPT technology for CNVs screening is more widely accepted in China. As early as 2015, R. Li et al. reported the clinical performance of NIPT for CNVs detection using 117 cases with known fetal CMA results. The sensitivity and specificity of NIPT for CNVs >1 Mb in that study were 61.1% and 95.0%, respectively (R. Li et al., 2016). Similarly, our center conducted a related survey, in which >80% of respondents expressed a preference for NIPT to report CNVs, even when the PPV is not high. Therefore, we included CNVs in our NIPT results and were constantly striving to improve the accuracy of the test.

A recent literature review showed that the PPV of NIPT for CNVs ranged 3%–100% (Zaninović et al., 2022). However, the studies with a PPV >80% in this literature were almost exclusively prospective studies of NIPT in samples with known CNV outcomes, which is certainly not consistent with clinical practice. Therefore, here we summarize the literature on the clinical applications of NIPT for CNVs (Table 4), focusing on cases where NIPT was performed without prior knowledge of the fetal outcome. Additionally, we excluded studies with fewer than 10,000 screened cases or fewer than 10 confirmed cases. The PPV in these studies ranged from 7.4% to 74.20%, with the majority of the study data originating from China and United States. The results of this study revealed that the PPV of NIPT for CNVs was 57.14%, which was higher than that reported in most studies. Only one study, with a single case having a read count of 30 million, and one study with a detection range of CNVs greater than 7 Mb reported a PPV higher than ours (Fiorentino et al., 2017; Rafalko et al., 2021). Some of the studies mentioned the fraction of cffDNA, with the mean or median values of the cffDNA fraction ranging from 7.89% to 12.57% and PPV ranging from 14.89% to 51.22% (Martin et al., 2018; Liang et al., 2019; Pei et al., 2020; Chen et al., 2021; Lai et al., 2021; Xue et al., 2022; Yang et al., 2022). Before we improved the library construction method, the cffDNA fraction (13.76%) and the PPV (28.57%) were comparable to the values reported in these studies. After the improvement, both the cfDNA fraction and PPV surpassed the values reported in those studies.

**TABLE 4:** Clinical applications of NIPT for PPV of CNVs.

Study	Country	Detection of CNV type	Number of reads	NIPT cases	CNVs positive cases	TP	FP	PPV (%)	cffDNA fraction (%)
Petersen et al. (2017)	United States of America	microdeletions (common)		/	52	7	45	13.40	
Martin et al. (2018)	United States of America	microdeletions (common)	>3.2 M	74938	283	24	129	15.70	10.5 (3.8–50.0)^a^
Schwartz et al. (2018)	United States of America	microdeletions (common)		/	349	25	310	7.40	
Fiorentino et al. (2017)	Italy	CNVs	30M	12114	30	8	5	61.54	
Liang et al. (2019)	CHINA	CNVs	20M	94085	163	49	71	40.80	10.8 (3.0–47.6)^b^
Chen et al. (2019)	CHINA	CNVs		42910	109	20	49	28.99	
Pei et al. (2020)	CHINA	CNVs	10.59M	36599	330	21	120	14.89	7.89 (6.18–10.76)^c^
Liu et al. (2020)	CHINA	CNVs		42924	56	11	27	28.95	
Rafalko et al. (2021)	United States of America	CNVs >7 Mb and common microdeletions		86902	490	181	63	74.20	
Chen et al. (2021)	CHINA	CNVs	0.1× coverage	34620	57	21	20	51.22	9.94 (3.48–50.19)^a^
Lai et al. (2021)	CHINA	CNVs	>3M	86193	13	4	8	33.33	12.57^d^
Wang et al. (2024)	CHINA	CNVs	6 M	135981	87	10	34	22.70	
Cai et al. (2023)	CHINA	CNVs		52855	74	23	47	32.90	
Yang et al. (2021)	CHINA	CNVs	3M	42969	250	61	136	30.96	
			8M	7710	123	41	53	43.61	
Xue et al. (2022)	CHINA	CNVs	8M	31256	221	78	125	38.42	11.2 (4.0–48.3)^b^
Zheng et al. (2022)	CHINA	CNVs		20538	38	15	23	39.47	
Yang et al. (2022)	CHINA	CNVs	15.45M	19086	170	40	73	35.42	8.24 (5.6–12)^c^
Liu et al. (2022)	CHINA	CNVs		/	52	21	31	40.40	
Shi et al. (2021)	CHINA	CNVs		36970	54	27	27	50.00	
Wang et al. (2021)	CHINA	CNVs		38974	95	25	26	49.02	
Chen et al. (2022)	CHINA	CNVs	20M	39580	/	30	42	41.70	
Zou et al. (2022)	CHINA	CNVs	20M	23116	31	15	14	51.72	

^a^ Mean (range).

^b^ Median (range).

^c^ Median (interquartile range).

^d^ 90.76% cases (n = 78,231) = ∼12.57%.

Compared to most other studies that focused solely on microdeletion, our study also investigated the detection efficacy of microduplication. Among the confirmed cases in this study, 33.70% were microduplication, showing a similar PPV to that of microdeletion. The PPV of microduplication (59.62%) was slightly higher than that of microdeletion (55.96%). We observed that for abnormalities smaller than 20 Mb, there was a positive correlation between abnormality size and PPV, with larger abnormalities showing higher PPV. However, when the abnormality size was ≥20 Mb, the PPV was notably reduced. Similar reductions in PPV for abnormalities ≥10 Mb have been also noted in other studies (Chen et al., 2019; Wang et al., 2021; Yang et al., 2021; Wang et al., 2022). This is contrary to reports suggesting that NIPT is more effective in predicting large segmental abnormalities (Yin et al., 2015), and that the PPV was much higher for cases with CNV ≥10 Mb (32%) compared to those with CNV <10 Mb (19%) (Liang et al., 2019). We suggested that the observed discrepancy may arise from the high PPV of 10–20 Mb (as high as 70.59% in our study) and the small number of cases. In contrast, the above-mentioned reports collectively categorized 10–20 Mb and ≥20 Mb abnormalities as ≥ 10 Mb. The lower PPV for CNVs >20 Mb may be resulted from the high risk of fetal loss in early pregnancy, as larger CNVs may render the fetus more susceptibility to abnormalities that could result in fetal loss before NIPT is performed (Wang et al., 2020; Wu et al., 2021; Zeng et al., 2023).

The cffDNA fraction and sequencing depth have been identified as the main factors affecting the detection efficiency of NIPT. It has been reported that the increase of resolution though increasing sequencing throughput yields diminishing returns. But increasing the fraction of cffDNA is a more effective option, as the improvement gained is at the molecular level rather than being a result of algorithmic adjustments (Welker et al., 2021). At the molecular level, it has been observed that fetal DNA fragments are shorter than maternal DNA fragments (Qiao et al., 2019). Currently, the primary method for increasing the fraction of cffDNA involves screening DNA fragments using magnetic beads and agarose gels. Pescia, G et al. showed a PPV of up to 70% for CNVs by NIPT after screening DNA fragments using gel electrophoresis (Pescia et al., 2017). In our study, the PPV of NIPT for CNVs (59.86%) was also high after increasing the concentration of cffDNA through magnetic bead. This suggested that cffDNA fraction played a crucial role in the detection efficacy of CNVs. Increasing the cffDNA fraction can enhance the sensitivity and PPV of NIPT for detecting CNVs. Related studies have also reported that cffDNA fraction is an important factor affecting the detection of CNVs by NIPT (Lo et al., 2016; Avram et al., 2021). Our review of false-negative cases suggested that increasing the cffDNA fraction was more effective than increasing Reads in improving CNV detection efficacy. One study also reported that false-negative cases with lower cffDNA fraction achieved positive results after increasing the cffDNA fraction (Hu et al., 2019). Expanding the application of non-invasive prenatal testing (NIPT-Plus) by increasing sequencing throughput is currently adopted by many testing organizations to enhance CNVs detection. However, it comes with a cost, being 30%–40% more expensive compared to regular NIPT. The NIPT method in our study, with a higher cffDNA fraction (18.44% ± 5.77%), demonstrated a comparable PPV to that reported in related studies, and it did not incur additional costs like NIPT-Plus for CNV detection (Wang et al., 2021; Xue et al., 2022). Some studies have pointed out that the detection efficacy for CNVs above 1 Mb is high when the cffDNA fraction is up to 20% (Li et al., 2016; Avram et al., 2021; Wang et al., 2022). In our study, the PPVs of NIPT for several MMs (such as DiGeorge syndrome, X-linked ichthyosis, 17p12 duplication syndrome, etc.) with sizes around 1 Mb were also high (64.71%). Therefore, we believe that NIPT is now sufficiently capable of detecting CNVs above 1 Mb and can alleviate the economic burden on pregnant women compared to NIPT-Plus.

Our characterization of the positive results revealed that the age of cases with positive results for CNVs by NIPT was much lower than that of cases with chromosomal aneuploidy anomalies. The percentage of advanced age (20.64%) was much lower than that of advanced age in the common trisomy (40.62%), confirming that the occurrence of CNVs is independent of maternal age (Avram et al., 2021; Wu et al., 2021). In addition, we found that some of the abnormalities observed in the fetuses with CNVs were inherited from mothers with intellectual disability, suggesting a genetic link in the production of some fetuses with CNVs (Avram et al., 2021; Cao et al., 2023). In our study, we observed that 83.41% of cases positive for common trisomy exhibited obvious clinical symptoms, whereas only 65.12% of cases positive for CNVs showed such symptoms. This suggested that more than one-third of CNVs may go undetected during screening due to the absence of obvious clinical symptoms. As a developing country, a significant portion of Chinese residents do not have high incomes, and coupled with an inadequate social security system, many families find it challenging to bear the enormous burden of caring for a child with disabilities. Presently, conventional screening methods such as serologic screening and ultrasonography lack in a high detection rate for CNVs. Moreover, invasive diagnostic methods not only entail a certain risk of pregnancy loss (Beta et al., 2018) but also come with a high cost, rendering them unsuitable for large-scale implementation. NIPT as a screening method has high sensitivity and specificity for CNVs, higher detection rate and PPV than other screening methods, and is safer and cheaper than invasive diagnostic methods.

Our study, while informative, has several limitations. We did not conduct placental examinations to validate false positive results, nor did we explore the reasons behind them. These are aspects we aim to investigate in our future studies. Additionally, we must always consider the inherent challenges posed by the nature of cell-free DNA (cfDNA).

## 5 Conclusion

Because of cultural differences and economic conditions, most Chinese pregnant women would like to know whether their children have health-affecting chromosome microdeletion/duplication during pregnancy and would like to have a safe, accurate, and cost-effective method to detect these abnormalities. Our study showed that NIPT has high sensitivity and specificity for CNVs, and the effect of cffDNA fraction on the detection efficacy may be greater than that of Reads, increasing the cffDNA fraction could improve the detection efficacy of NIPT. Therefore, we recommended utilizing NIPT for screening fetal CNVs in clinical work, and future research should focus on improving cffDNA enrichment methods.

## Data Availability

The original contributions presented in this study were publicly available. This data can be found in Figshare https://doi.org/10.6084/m9.figshare.25909822.

## Supplementary material

SUPPLEMENTARY FIGURE S1 Maternal age multiple comparisons of different cases.

SUPPLEMENTARY FIGURE S2 The PPV for CNVs of varying sizes.

SUPPLEMENTARY TABLE S1 Summary of 281 positive results with CNVs.

SUPPLEMENTARY TABLE S2 Comparison of whether CNVs and common trisomies have clear clinical indications.

SUPPLEMENTARY TABLE S3 Comparison of PPV between microdeletion and microduplication cases.

SUPPLEMENTARY TABLE S4 Comparison of cffDNA fraction, Reads and GC concentration before and after the improvement.

SUPPLEMENTARY TABLE S5 Review results of false-negative case.
