# Supplementary material for: The performance evaluation of NIPT for fetal chromosome microdeletion/microduplication detection: a retrospective analysis of 68,588 Chinese cases
Source: Front Genet. 2024 Jun 7;15:1390539. doi: 10.3389/fgene.2024.1390539 (PMC11190309; doi:10.3389/fgene.2024.1390539)
Supplement: Supplementary file 2 [file Table1.DOCX]

Supplementary Material

The performance evaluation of NIPT for fetal chromosome microdeletion/microduplication detection: a retrospective analysis of 68,588 Chinese cases

Shichun Shen^1^, Haimei Qi^2^, Xianping Yuan^3^, Jinhui Gan^1^, Junkun Chen^1*^ and Jungao Huang^1*^

*** Correspondence:**

Junkun Chen
1908193553@qq.com

Jungao huang
jungaohuang@sina.com

# Supplementary Figures and Tables

**Supplementary Table S1** Summary of 281 positive results with CNVs.

The detailed table is attached.

**Supplementary Table S2** Comparison of whether CNVs and common trisomies have clear clinical indications.

| **Index** | **Clear clinical indications** | | **Total** | **Rate** | **Asymptotic Significant** |
| --- | --- | --- | --- | --- | --- |
|  | **Yes** | **No** |  |  |  |
| CNVs | 185 | 96 | 281 | 65.84% | <0.001 |
| Common trisomy | 357 | 71 | 428 | 83.41% |  |
| Total | 542 | 167 | 709 | 76.45% |  |

Using the Pearson Chi-Square tests, the rates difference was statistically significant at the 0.05 level.

**Supplementary Table S3** Comparison of PPV between microdeletion and microduplication cases.

| **Index** | **Positive** | **Negative** | **Total** | **PPV** | **Asymptotic Significant** |
| --- | --- | --- | --- | --- | --- |
| Deletion | 61 | 48 | 109 | 55.96% | 0.661 |
| Duplication | 31 | 21 | 52 | 59.62% |  |
| Total | 92 | 69 | 161 | 57.14% |  |

Using the Pearson Chi-Square tests, the rates difference was statistically significant at the 0.05 level.

**Supplementary Table S4** Comparison of cffDNA fraction, Reads and GC concentration before and after the improvement.

| **Index** | **Number** | **Independent Samples Test** | | | | | |
| --- | --- | --- | --- | --- | --- | --- | --- |
|  |  | **CffDNA fraction (%)** | | **Reads(M)** | | **GC concentration (%)** | |
|  |  | **Mean** | **SD** | **Mean** | **SD** | **Mean** | **SD** |
| pre-improvement | 12885 | 13.76 | 5.68 | 4.07 | 0.49 | 41.09 | 0.69 |
| post-improvement | 55703 | 18.44 | 5.80 | 4.09 | 0.68 | 41.18 | 0.76 |
| Significant | | <0.001 | | >0.05 | | >0.05 | |

The means difference was statistically significant at the 0.05 level.

**Supplementary Table S5** Review results of false-negative case.

| **Index** | **cffDNA fraction (%)** | **Reads(M)** | **GC concentration (%)** | **Average read length(bp)** | **Result** |
| --- | --- | --- | --- | --- | --- |
| Initial test | 11.7 | 4.58 | 41.3 | 163 | negative |
| triple-Reads review | 11.3 | 10.17 | 42.0 | 160 | negative |
| rebased library review | 19.7 | 4.52 | 40.3 | 143 | chr5: del:0-9M |
