# Supplementary figures and images for: The performance evaluation of NIPT for fetal chromosome microdeletion/microduplication detection: a retrospective analysis of 68,588 Chinese cases
Source: Front Genet. 2024 Jun 7;15:1390539. doi: 10.3389/fgene.2024.1390539 (PMC11190309; doi:10.3389/fgene.2024.1390539)

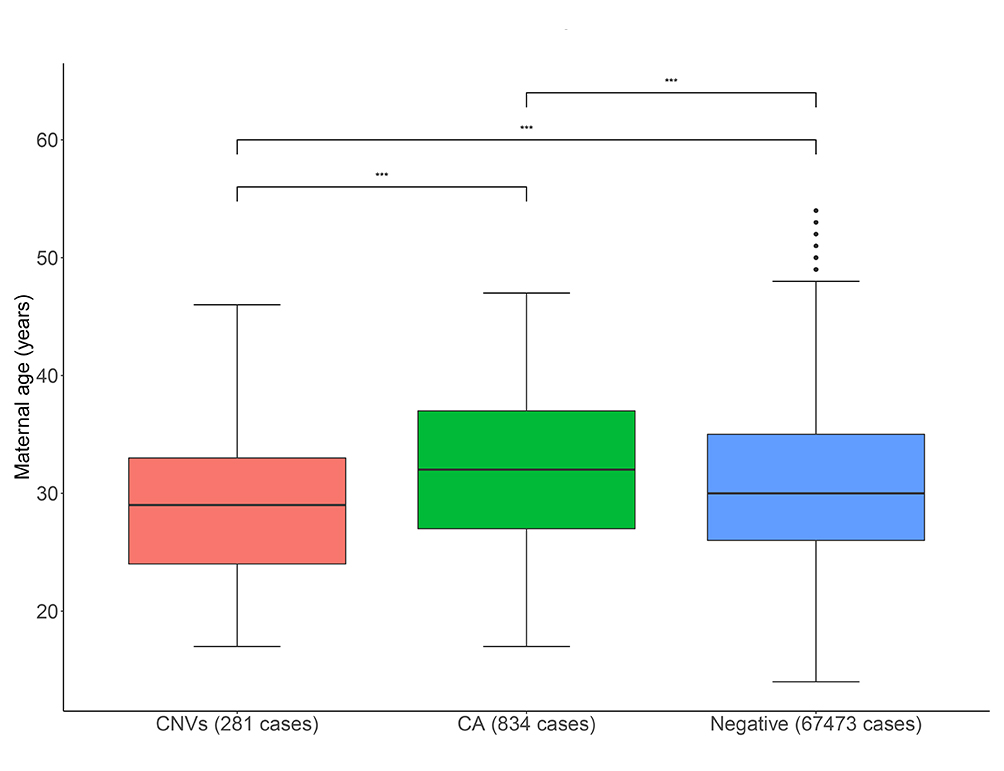

Supplement: Supplementary file 3 [file Image1.JPEG]

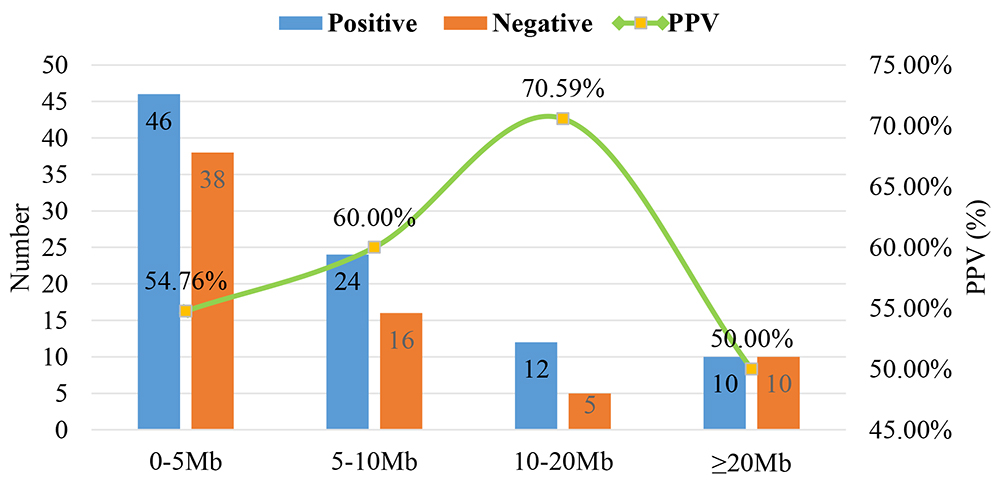

Supplement: Supplementary file 4 [file Image2.JPEG]
